# Supplementary material for: Assessing Leadership Capacity in the Food System: The Issue Leadership Scale
Source: Foods. 2023 Oct 12;12(20):3746. doi: 10.3390/foods12203746 (PMC10606580; doi:10.3390/foods12203746)
Supplement: Supplementary file 1 [file foods-12-03746-s001.zip › foods-2664153-supplementary.pdf]

foods MDPI

Supplementary Materials

Issue Leadership Importance Scale

"Please indicate how important each of the following behavior categories are to success in your current position by marking the appropriate circle."

1. Action (advocacy, taking leadership roles, etc.)
2. Change (acting as a change agent, innovating, etc.)
3. Communication (listening, media relations, etc.)
4. Critical Thinking, Strategic Planning & Visioning (decision making, systems thinking, etc.)
5. Interpersonal Traits and Characteristics (confidence, empathy, ethics, etc.)
6. Leadership Skill (conflict management, enabling others, etc.)
7. Leadership Process (collaboration, team dynamics, etc.)

Note: 1—Not at all important, 2—Very Unimportant, 3—Neither Important or Unimportant, 4—Very Important, 5—Extremely Important

Issue Leadership Skill Scale

"Please indicate how skilled you believe you are at each of the following behavior categories."

8. Action (advocacy, taking leadership roles, etc.)
9. Change (acting as a change agent, innovating, etc.)
10. Communication (listening, media relations, etc.)
11. Critical Thinking, Strategic Planning & Visioning (decision making, systems thinking, etc.)
12. Interpersonal Traits and Characteristics (confidence, empathy, ethics, etc.)
13. Leadership Skill (conflict management, enabling others, etc.)
14. Leadership Process (collaboration, team dynamics, etc.)

Note: 0—Not Applicable (no knowledge) 1—Fundamental Awareness (basic knowledge), 2—Novice (limited experience), 3—Intermediate (practical application), 4—Advanced (applied theory), 5—Expert (recognized authority)
